# Athletes Perceive Weighted Baseballs to Carry a Notable Injury Risk, yet Still Use Them Frequently: A Multicenter Survey Study

**DOI:** 10.5435/JAAOSGlobal-D-21-00306

**Published:** 2022-09-09

**Authors:** Austin G. Cross, Lafi S. Khalil, Alexander J. Swantek, Vincent A. Lizzio, Alexander C. Ziedas, Christopher L. Camp, Peter N. Chalmers, Karch Smith, Sarah E. Chaides, John D. Rexroth, Eric C. Makhni

**Affiliations:** From the Henry Ford Hospital (Dr. Cross, Dr. Khalil, Dr. Swantek, Dr. Lizzio, Dr. Ziedas, Ms. Chaides, Mr. Rexroth, and Dr. Makhni), Department of Orthopaedic Surgery, Detroit, MI; the Department of Orthopedic Surgery and Sports Medicine (Dr. Camp), Mayo Clinic, Rochester, NY; and the Department of Orthopedic Surgery (Dr. Chalmers and Smith), University of Utah, Salt Lake City, UT.

## Abstract

Weighted baseball use in throwing programs is widespread; however, their use remains controversial. Prior research shows that weighted baseball programs can increase ball velocity but potentially increase throwing arm injuries. This study aims to ascertain perceptions of weighted baseballs among elite baseball players.

**Methods:**

A created online survey questioned common practices, throwing regimens, injury risk factors, and weighted baseball program use. The questions were modeled to ascertain the perceptions of elite baseball players to understand their experience with weighted baseballs. Descriptive statistical analysis was conducted.

**Results:**

Three hundred seventy-six baseball players with a mean age of 20 ± 2 years completed the survey; 64% of the players (239/376) were pitchers. 71% (267/376) reported the use of weighted baseballs. Of those, 75% (199/267) thought it made them a better player. Overall, 73% (275/377) thought weighted baseballs are a risk for injury. 17% (46/267) attributed their injury to using weighted baseballs. Overall, participants reported a mean 72% ± 30% likelihood of future weighted baseball use.

**Conclusion:**

Most of the participating elite adult baseball players reported prior weighted baseball use with a corresponding improvement in pitching performance despite a perceived increased injury risk. Nearly 20% of the players attributed pain or injury to weighted baseball use. Moreover, the players surveyed intend to continue using weighted baseballs because of the perceived performance benefit.

The use of weighted baseballs during throwing programs is widespread across ages and competitive levels, with the goal of improving throwing performance (ball velocity and accuracy).^1^ Weighted baseball training programs commonly involve the use of various overweight, underweight, and standard baseballs (5 ounce) either alone or in concert with one another at varying distances, throwing stances, and throwing techniques.^2^ These baseballs are commercially available, with weights commonly ranging from 2oz to over 32oz. Weighted baseball training programs are especially common among pitchers, where maximizing ball velocity often plays a notable role in player success and advancement.^2,3,4,5,6^ Although their use as a training modality has increased in recent years, there remains controversy surrounding the efficacy and potential risks. Proponents claim that weighted baseballs can be safely used to increase arm speed and strength and ultimately increase ball velocity and that injuries related to weighted baseball training can often be attributed to inappropriate program design.^7^ Those advising against the use of weighted baseballs argue that there is insufficient evidence supporting their safe use or that they should only be used in select individuals with certain criteria met (skeletal maturity, proper mechanics, appropriate supervision, etc.). In fact, a recent study suggested that weighted baseball programs may be associated with increased risk of throwing arm injuries.^8^

Currently, there are no established universal guidelines based on scientific evidence that outline how to implement weighted baseballs safely and effectively into a training program. Given the concerning increase in shoulder and elbow injuries in baseball players,^9,10,11,12,13,14^ it is prudent for orthopaedic surgeons and other sports medicine practitioners to play an active role in guiding the safe implementation of these weighted baseball programs. However, before standard guidelines can be established, it is important to first understand the prevalence and common practices and perceptions of weighted baseball throwing programs among players.

The purpose of this study was to understand the perceptions of weighted baseball training programs in adult baseball players, particularly in relation to perceived advantages and disadvantages, history of use, and risk factors for injury. We hypothesize that most of the players will be familiar with weighted baseballs and perceive a performance benefit with their use. In addition, we hypothesize that any perceived risk of injury associated with weighted baseballs would deter competitive athletes from future use of weighted baseballs.

## Methods

This was a multicenter survey study among three institutions conducted between August and November 2020. Approval for study conduction and data sharing was obtained from the institutional review boards of each of the three involved institutions before the initiation of this study.

The study authors created a survey designed to assess the perceptions of elite baseball players regarding the use and risks of weighted baseballs in structured throwing programs (Appendix A, http://links.lww.com/JG9/A235**)**. Question formatting included binary yes/no options, checkboxes, multiple choices, visual analog scales, and fill-in-the-blank responses. The survey was distributed by each participating institution to their affiliate baseball programs at the collegiate and professional levels. Participants of the survey electronically received an online link, which included a consent form before the initiation of the survey. Pitchers, in particular, were targeted for participation because of the high prevalence of weighted baseball use in this group; however, all positional players were welcomed to participate. Survey response data, which were anonymous, were stored electronically using the Research Electronic Data Capture, a secure and HIPAA-compliant data management and collection tool developed by Vanderbilt University (Nashville, TN).^15^ The online survey used conditional branching to collect the most accurate and appropriate responses from each participant.

### Study Population

Initial questions included demographical information about age, height, weight, throwing arm, and ethnicity. Participants were asked to identify their primary sport and their primary baseball position. Players were asked about their personal injury and surgical history whether they have ever experienced an injury or surgery to their throwing arm that caused them to miss practice or games. Additional information, including date of injury/surgery and specific type of injury/surgery, was also collected. A visual analog scale was then used to ask how much pain the player currently experiences in their throwing arm while throwing.

### Perceived Advantages and Disadvantages

Participants reported their familiarity with weighted baseball training programs. To assess perceived benefits of a weighted baseball training program, participants were asked to rank possible benefits (injury prevention, increased velocity, improved accuracy, and improved throwing mechanics or range of motion) according to the benefit gained from their use. Similarly, participants were then asked to rank perceived disadvantages and risks associated with weighted baseballs, such as the cost/expense or increased injury risk.

### History of Use

Participants reported their use of weighted baseballs, including overweight and/or underweight, and the frequency of their training regimen. Details of when they initiated weighted baseball training, how they were introduced to it, and their likelihood of continuing to use it were also collected. Players who have used weighted baseballs were asked whether the training program helped them become a better pitcher and how they improved specifically (ball velocity, accuracy, etc.). Players were also asked whether they had experienced any problems with the use of weighted baseballs and the details regarding these problems (pain, injury, decreased performance, etc.).

### Risk Factors for Injury

Players were asked whether they thought weighted baseball training programs could cause injury and to what specific area (shoulder, elbow, wrist, and hand) the injury would most likely occur. To gauge a general understanding of knowledge related to injuries, players were asked what variables were related to throwing injuries, and what variables were the most important risk factors for injury (pitch number, pitch type, rest time, etc.). Finally, players were asked what the youngest appropriate age is for someone to start a weighted baseball training program and about the maximum weight of a baseball that is safe to throw during a training program.

### Statistical Analysis

Survey response data were downloaded from Research Electronic Data Capture, and descriptive statistics, in the form of counts, percentages, means, and standard deviations, were assessed for each survey response. Independent samples Student *t*-tests were conducted to identify notable differences between players who previously had throwing arm surgery and those who had not.

When analyzing responses, calculations were made using the whole population and/or subsets based on the question being answered. When population subsets were used, they were determined by responses to other questions in the survey, and subset percentages were reported according to the number of responses for the determining question(s). All analyses of significance used a level of 5% and were conducted using SPSS software (IBM Released 2020. IBM SPSS Statistics for Windows, Version 27.0: IBM.).

## Results

A total of 376 players were surveyed, 323 (86%) of whom reported familiarity with weighted baseball training programs. The mean ± SD participant age was 20 ± 2 years, comprising players in college (342) and professional leagues (34). Players reported a mean throwing arm pain intensity of 17 ± 22 (median = 10) on a 0 to 100 scale (Table 1). Most of the participants (n = 239/376, 64%) were pitchers (Table 1). Additional demographic information is presented in Table 1.

**Table 1:** Participant Demographics

	Mean ± SD	Range
Age, y	20 ± 2	17-39
Height, m	1.87 ± 0.06	1.68-2.03
Weight, kg	90 ± 9	66-113
BMI, kg/m^2^	26 ± 2	19-33
Pain with throwing, out of 100	17 ± 22	0-100
		Participants (%)
Primary sport		
	Baseball	329 (88)
	Other	47 (12)
Primary position		
	Pitcher	239 (64)
	Catcher	22 (6)
	Infield (1B, 2B, 3B, SS)^a^	41 (11)
	Outfield	27 (7)
	Other	47 (12)
Dominant hand		
	Right	289 (77)
	Left	87 (23)
Level of play		
	College	342 (91)
	Professional	34 (9)
Injury history		
	Missed game because of throwing arm injury	174 (46)
	Surgery on the throwing arm	51 (14)

Of all participants, 267 (71%) had engaged in a weighted baseball training program, with 89% of the participants (238/267) using both overweight and underweight baseballs. Each player used a mean of 4.2 ± 1.9 different overweight baseballs in their training programs. The most commonly used overweight baseballs were 6oz and 7oz, representing 17% and 15% of overweight baseball responses, respectively. Each player used a mean of 1.7 ± 0.7 different underweight baseballs in their programs, with 4oz (44%) and 3oz (38%) being the most commonly used.

Among users of weighted baseballs, the most frequently reported usage was 3 to 5 times per week (n = 144/266, 38%), with <2 times per week being the least frequently reported (n = 54/266, 14%). For underweight baseballs, most players reported (n = 110/239, 29% of the players) using them less than two times per week, with 8% of the players (n = 29/239) using them daily.

Overall, players perceive the greatest benefit of weighted baseball use to be improved or increased pitch velocity, with the least being improved or increased control/accuracy (Table 2). Of the players who actually used a weighted baseball program, 75% (199/267) reported that their use helped make them a better player, with perceived improvement in a mean of 2.2 ± 1.0 of the listed domains—ball velocity, accuracy/location, ball movement, arm strength, and others. The most common improvements were in velocity (86%) and arm strength (79%). Accuracy/location (26%) and pitch movement (21%) were also noted as improved domains (Table 3).

**Table 2:** Perceived Benefits and Risks of Weighted Baseballs Among All Respondents

Benefits					
Increased/improved	Least 1	2	3	4	Greatest 5
Pitch velocity	10	13	40	61	*177*
Control/accuracy	*158*	81	36	19	4
Mechanics/techniques	15	64	75	82	68
Range of motion	35	75	89	87	27
Injury prevention	73	69	73	71	29
Risks					
Biggest downside	Respondents	Percentage (%)			
Increased injury risk	165	51			
Costs/expense	70	22			
No risks	71	22			
Other	17	5			

**Table 3:** Perceived Improvements From Weighted Baseball Use

	Participants	Percentage
Did weighted baseball use make you a better player?		
Yes	199	75
No	68	25
If yes, how did you improve?		
Pitch velocity	171	86
Arm strength	158	79
Accuracy/location	51	26
Pitch movement	41	21
Other	9	5%

Players most commonly cited not having enough rest between throwing days (72%) being related to throwing injuries, with throwing while in pain (76%) being the most important risk factor for a throwing injury, in general (Table 4). Furthermore, weighted baseball training programs were thought to be a risk factor for injury by 73% of all players (275/376) and 85% of the players (226/267) who have participated in a weighted baseball program. In addition, 17% of the players (46/267) who participated in a weighted baseball program attributed a throwing problem they experienced in their use of weighted baseballs. Of those players, 72% (33/46) experienced pain or injury to their throwing arm.

**Table 4:** Perceptions of Throwing Injuries, Risk Factors, and Maximum Safe Baseball Weight

	Participants	Percentage
Throwing practices related to throwing injuries		
Not enough rest days between throwing	270	72
No. of pitches/balls thrown	264	70
No. of games pitched/played	159	42
Types of pitches thrown (fastballs, curveballs, change-up, slider, etc.)	88	23
Speed of pitches thrown	77	21
Most important risk factors for throwing injuries		
Throwing while having pain	286	76
Overuse	257	69
Throwing while having pain	242	65
Types of pitches thrown (fastballs, curveballs, change-up, slider, etc.)	239	64
No. of pitches/balls thrown	217	58
No. of games pitched/played	114	30
Types of pitches thrown (fastballs, curveballs, change-up, slider, etc.)	49	13
Speed of pitches thrown	42	11
Throwing balls	10	3
Throwing strikes	5	1
Throwing balls	3	1
Throwing strikes	2	1
Maximum weight to train with		
5-6oz	12	3
7oz	23	6
8oz	28	7
9oz	22	6
10oz	31	8
11oz	24	6
12oz	46	12
≥16oz/1lb	133	35
Other/no response	57	15

**Figure 1:**
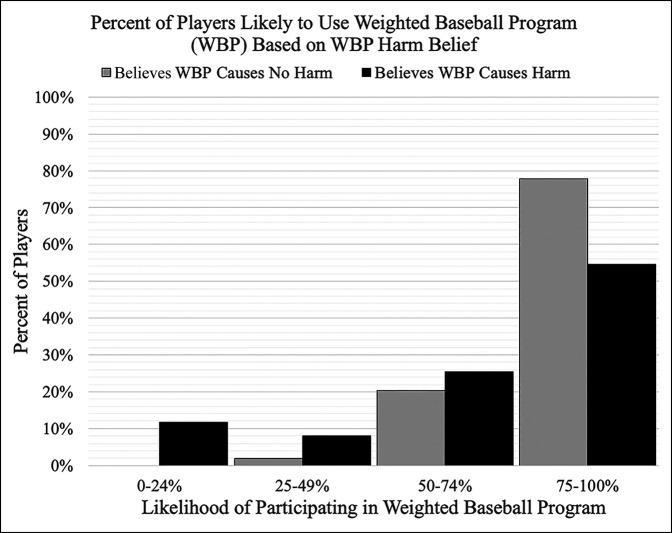
Graph showing the percentage of players likely to use a weighted baseball program (WBP) in the future based on their belief of whether a WBP can cause harm. Players indicated their willingness to participate in a WBP program on a scale of 0 to 100%. There were a total of 271 players thinking that a WBP could cause harm and 54 thinking that WBPs did not cause harm. Players thinking that WBPs caused no harm had a mean of 89% ± 16% and a median of 98.5% likelihood while players thinking that WBPs caused harm had a mean of 69% ± 31% with a median of 75% likelihood of future WBP use. The most common responses for both groups were in the 75% to 100% range, with 80% of the players who think WBPs cause harm and 98% of the players who do not think WBPs cause harm reporting a 50% to 100% likelihood to participate in a WBP in the future.

**Figure 2:**
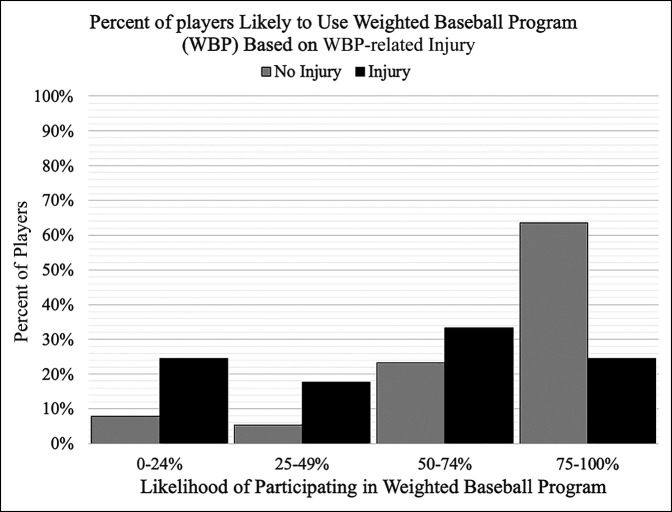
Graph showing the percentage of players likely to use a weighted baseball program (WBP) again in the future based on whether the player attributes an injury to prior WBP use. Players indicated their willingness to participate in a WBP program in the future on a scale of 0 to 100. There were a total of 280 players with no injury attributable to WBP use and 45 with an injury attributed to a previous WBP. Players without injury had a mean of 76% ± 28% and a median of 84.5% likelihood of future use while players attributing injury to WBP use had a mean of 49% ± 33% with a median of 50% likelihood of future WBP use.

Overall, participants reported a mean 72% ± 30% (median = 80%) likelihood of future weighted baseball use. Regardless of player perception on the harmful potential of weighted baseballs, the most commonly reported likelihood of future use was 75% to 100% (Figure 1). Similarly, there was no significant difference in future likelihood of weighted baseball use between players who previously had surgery on their throwing arm (66% ± 36%) and those who never had surgery on their throwing arm (72% ± 30%) (*P* = 0.245). From the 46 players who reported a throwing-related problem because of weighted baseball programs, the reported likelihood response mean was 49% ± 33%, with 57% of the players indicating a 50% to 100% likelihood (Figure 2). When asked, the participants thought that an acceptable age for players to begin using weighted baseball programs was a mean of 15 ± 2 years and a maximum ball weight of less than 1 pound was preferred by 65% of the players (Table 4).

## Discussion

Despite their prevalence, little is understood about weighted baseballs regarding actual performance improvement and injury risk. The purpose of this study was to understand the perceptions of weighted baseball programs among active adult baseball players. The results of this study indicate that most of the players perceive weighted baseballs not only to be beneficial for pitching performance but also to be risk factors for throwing arm injuries. Nearly 20% of the survey responders attributed their arm injury to the use of weighted baseball programs. This study highlights the fact that elite athletes are willing to assume an increased injury risk associated with weighted baseball use with hopes of increasing their performance. Knowing this, orthopaedic surgeons should focus educational efforts and future research into developing protocols on how to implement weighted baseballs effectively and safely into athlete's training regimens.

Most of the respondents in this survey study cited an increase in ball velocity as the greatest perceived benefit of a weighted baseball program, which has been investigated by several previous studies.^8,16,17^ In a recent meta-analysis of weighted baseball utilization, Caldwell et al^2^ identified a resultant increase in ball velocity; however, the authors noted that the overall quality of the studies was poor (because of considerable variation in the training protocols, weight of baseballs used, player level, etc.). Similarly, Erickson et al^18^ found that pitchers experienced a mean 4.8-mile per hour increase in maximum ball velocity over a 15-week program using underweight baseballs. Although none of the 44 pitchers who completed the protocol reported injuries, it should be noted that this study did not include a control group nor a group that used overweight baseballs. A similar study by Yang et al^19^ found notable increases in maximal ball velocity during a 10-week training program using only underweight baseballs. A previous study by Okoroha et al^20^ investigated the effect of ball weight on medial elbow torque using 3oz to 6oz baseballs, finding a notable increase in elbow torque with each 1oz increase in baseball weight. They also found that the players even preferred throwing with the lightest baseball (3oz) the most. The current investigation found little enthusiasm for the use of lightweight baseballs, which may highlight the lack of awareness around potential benefits—without reported injury risk—of underweight baseball training.^18,19,21^

Given the recent rise in the amount of injuries to the shoulder and elbow, especially the UCL, among overhead throwing athletes, the injury risk associated with the use of weighted baseballs is important to consider.^11,22,23,24,25^ A recent randomized controlled trial by Reinold et al^8^ investigated the effects of a 6-week weighted baseball training program using both overweight and underweight baseballs in 38 young male pitchers. Compared with the control group, the weighted baseball group was found to have notable increases in maximum ball velocity (3.3%) and shoulder external rotation (4.3%). The control group reported zero injuries, compared with a 24% injury rate in the weighted baseball group, which was especially concerning because their training protocol was considerably less aggressive (as defined by the length of program, baseball weights used, training volume, and frequency) than most of the programs commonly used. Overall, the body of literature examining injury rates in weighted baseball programs is limited, with many previous studies not commenting on the injury rates during the program or failing to follow up participants in subsequent seasons.^2^ Our investigation found that just less than 20% of the participants attributed a throwing problem to their weighted baseball program, with the majority being pain or injury to the throwing arm.

Increases in pitch velocity after weighted baseball programs are thought to be because of the resultant increase in shoulder external rotation.^1,8,26,27,28,29,30^ However, there may be harmful effects at the elbow because of these programs, leading to injuries to the UCL and other structures of the elbow.^7,20^ Numerous studies have correlated increases in pitching velocity with a corresponding increase in injury risk to the elbow.^31,32,33,34,35,36,37,38^ This may be because of an increase in the valgus force experienced at the ulnar collateral ligament.^34-36,39,40^ Therefore, elbow injury after participation in weighted baseball programs could be related to this short-term increase in velocity because of additional external rotation gained at the shoulder.

Current athlete perceptions may reflect the scarce and sometimes conflicting literature surrounding weighted baseballs. We recommend that elite athletes who choose to use weighted baseballs in their throwing programs understand the potential risks and do so cautiously under the direction of the team athletic trainers, physical therapists, and throwing coaches, with a low threshold to be evaluated by the team sports doctors with the development of any new symptoms related to throwing.

This investigation is not without limitations. First, the survey was completely anonymous and online, and thus, the authors could not directly oversee completion of the survey. However, each of the three institutions used preexisting relationships with their affiliate programs to distribute the survey. Second, it is possible that perceptions of weighted baseballs differ by geographical region. However, this was a multisite study with a large overall catchment area, with each participating institution spread out across the country. Third, there may be selection bias in that players who responded to the survey may be more interested in weighted baseball programs. This is especially true given the high level of competition of our respondents. It should also be noted that most of the participants were collegiate athletes, with a minority of professional participants. This may be explained by the larger proportion of collegiate athletes compared with professional players. In addition, owing to the method of survey dissemination as previously described, an accurate response rate cannot be calculated.

## Conclusion

In this survey study of elite adult baseball players, most of the participants reported prior use of weighted baseball training programs along with a corresponding improvement in pitching performance. This is despite perceiving an inherent increased injury risk associated with their use. Nearly 20% of the players attributed pain or injury to the use of weighted baseballs. Moreover, most of the players surveyed intend to continue using weighted baseballs in the future because of the perceived performance benefit.
